# Organic materials database: An open-access online database for data mining

**DOI:** 10.1371/journal.pone.0171501

**Published:** 2017-02-09

**Authors:** Stanislav S. Borysov, R. Matthias Geilhufe, Alexander V. Balatsky

**Affiliations:** 1 Nordita, Center for Quantum Materials, KTH Royal Institute of Technology and Stockholm University, Roslagstullsbacken 23, SE-106 91 Stockholm, Sweden; 2 ETH Institute for Theoretical Studies, ETH Zurich, 8092 Zurich, Switzerland; Oregon State University, UNITED STATES

## Abstract

We present an organic materials database (OMDB) hosting thousands of Kohn-Sham electronic band structures, which is freely accessible online at http://omdb.diracmaterials.org. The OMDB focus lies on electronic structure, density of states and other properties for purely organic and organometallic compounds that are known to date. The electronic band structures are calculated using density functional theory for the crystal structures contained in the Crystallography Open Database. The OMDB web interface allows users to retrieve materials with specified target properties using non-trivial queries about their electronic structure. We illustrate the use of the OMDB and how it can become an organic part of search and prediction of novel functional materials via data mining techniques. As a specific example, we provide data mining results for metals and semiconductors, which are known to be rare in the class of organic materials.

## Introduction

Computational materials science based on *ab initio* methods has a long history of more than half a century. Development of the density functional theory (DFT) framework in the 1960s by Hohenberg and Kohn [1] and Kohn and Sham [2] marked a clear breakthrough in providing an approach that is a standard tool in modern materials science [3]. In this connection, a variety of approaches to estimate the electron density have been considered and implemented [4–8]. By now, it has been established that the most prominent codes agree well in the calculation of physical quantities by showing errors comparable to the experiment [9]. Mostly, the calculations performed are focused on a particular material of interest and motivated, for example, by providing additional information to experiments (e.g. [10, 11]). This approach can be viewed as a “one-compound-at-a-time” analysis.

In the beginning of this century, the exponential growth of computational power and high demand for prediction of materials with target properties led to a new way of dealing with *ab initio* electronic methods referred to as materials informatics [12, 13]. This approach places the main effort on performing high-throughput computing and data mining [14–16] as well as the development of sufficient tools for that [17, 18]. One can call this approach an “aggregate informatics analysis”, where the properties of a single compound are captured approximately and main resource is placed on understanding global trends within the large datasets. Applications of this informatics-driven approach are wide-ranging and cover, for instance, the search for functional materials [19], topological insulators [20] or the prediction of stable crystal structures [21, 22]. Instead of recalculating material properties each time, results are made available in databases [23, 24].

Motivated by this new trend in materials informatics, we focus on organic and organometallic materials because of multiple reasons. Whereas inorganic materials are well-studied by the above described methods, organic crystals are investigated rarely. One of the main difficulties lies in the large-unit cells which can contain up to several hundred atoms. Even though reports on $O(N_{\text{atoms}})$ implementations are discussed in the literature [25, 26], usual DFT codes scale with $O(N_{\text{atoms}}^{2}\log N_{\text{atoms}})$ up to $O(N_{\text{atoms}}^{3})$ [27] leading to a high computational demand for large unit cells. New computational resources and modern code architectures have opened the path for such system sizes within the last decade [28, 29].

Organic crystals offer a high potential for technological applications [30, 31]. The main constituents of organic crystals are carbon, hydrogen, nitrogen, oxygen and, in rare cases, a low percentage of transition metal elements. This makes production of organics inexpensive and accessible in terms of raw materials. This potential for applications, utility and availability motivates the investigation of organic solar cells as realistic alternative to currently used cells based on inorganic semiconductors [32, 33]. Aside from application in organic solar cells, there are reports on *d*-wave superconductivity for the materials *κ*-(BEDT-TTF)_2_Cu(NCS)_2_ [34] and *κ*-(BEDT-TTF)_2_Cu[N(CN)_2_]Br [35]. Due to the softness, some materials show interesting conduction phenomena under high pressure, like the material *α*-(BEDT-TTF)_2_I_3_, where a tilted Dirac cone can be induced within the band structure close to the Fermi level [36]. The elastic properties of organic materials make them particularly suitable for various applications in flexible electronics [37, 38].

In this paper, we report on setting up a web database for organic crystals as a source for data mining projects promoting the *ab initio* investigation of organics and the prediction of organic functional materials. The database itself contains thousands (6461 at the time of writing) of calculated Kohn-Sham band structures. The implemented web interface allows for fast online search algorithms to identify materials with specified electronic properties.

**Fig 1:**
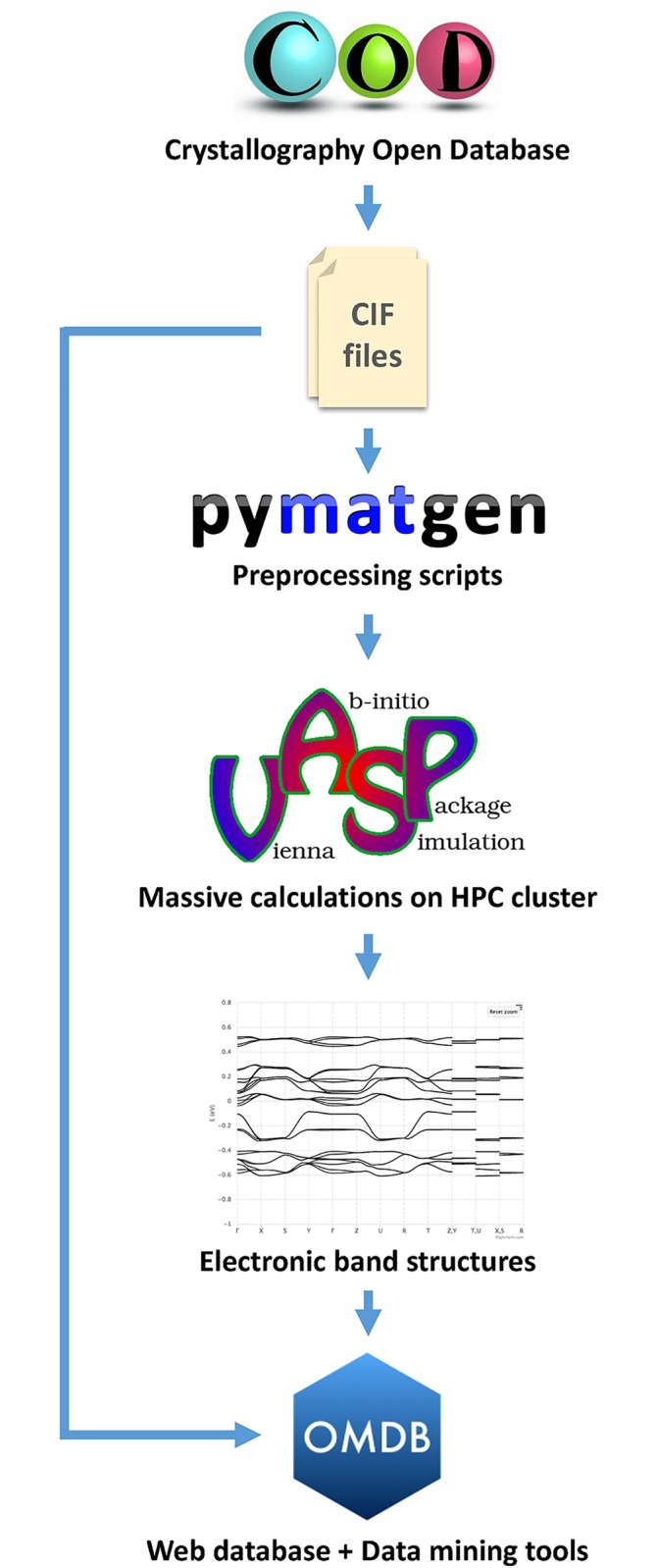
The OMDB data flow chart. Crystallographic data contained in the COD database in the CIF format is converted to DFT input by applying the Pymatgen package. DFT electronic structure calculations are performed using the VASP package. The DFT output (band structures and density of states) along with the basic crystallographic data from the CIF files are stored in the OMDB database, which also provides data mining tools to retrieve materials with specified by users electronic structure properties.

The overall data flow chart for the organization of the database is shown in Fig 1. Details are discussed throughout the paper.

The paper is organized as follows. In Materials and Methods, we describe the crystallographic data and DFT calculation details along with the OMDB software implementation. In Results, the OMDB web interface and capabilities for data mining are introduced. Examples of the database usage for mining of novel functional materials such as organic metals and semiconductors are provided in Discussion. Finally, the scope and capabilities are summed up in Conclusions, where we also discuss the current status of the OMDB database and its potential future improvements.

## Materials and methods

### Crystallographic data

The structural information for organic compounds were taken from the Crystallography Open Database (COD) [39–41] which is available online at http://crystallography.net. The COD provides structural information in the Crystallographic Interchange File/Framework (CIF) files [42].

**Fig 2:**
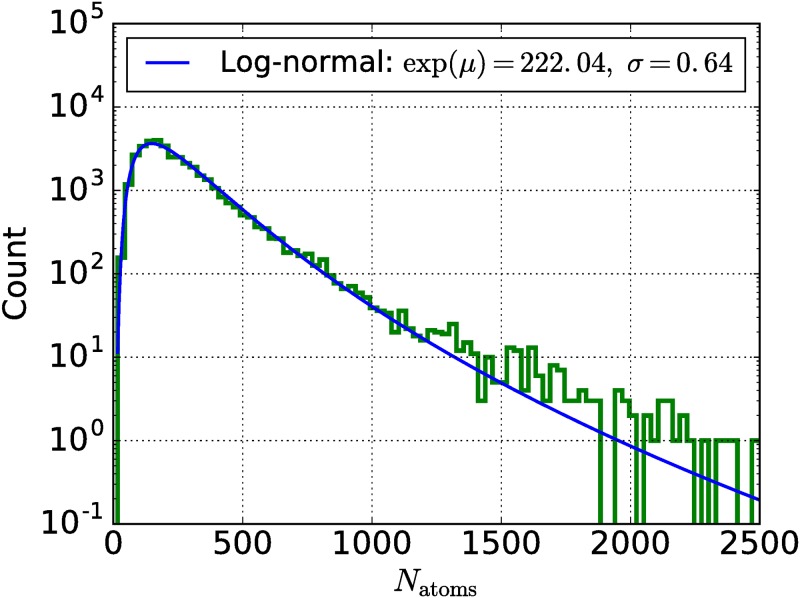
Histogram of number of atoms per unit cell for 37,941 organic compounds from four experimental organic chemistry journals contained within the COD database. Blue solid line denotes log-normal fit with a median value exp(*μ*) of 222.04 atoms and a standard deviation *σ* of 0.64 ln(atoms).

Although there are about 300,000 materials in the COD containing carbon, we decided to focus first on the 50,211 previously synthesized materials described in four dedicated experimental organic chemistry journals: “Organometallics” [43], “Organic Letters” [44], “Journal of Organic Chemistry” [45] and “Organic & Biomolecular Chemistry” [46]. However, it was not possible to do DFT calculations for all of them. Incomplete structures or structures with fractional occupation of ionic sides were excluded (12,270 structures or 24% of the initial data). For the remaining 37,941 materials, the main limitation lies on the polynomial complexity of DFT algorithms with respect to a number of atoms in the unit cell. Organic crystals have on average larger unit cells comparing to inorganic crystal structures. For illustration purpose, a histogram of *N*_atoms_ per unit cell for the considered materials is shown in Fig 2. The shape remarkably follows a log-normal distribution with median value of 222 atoms per unit cell.

To further elaborate on this point, we split the 37,941 input materials into four classes depending on *N*_atoms_ per unit cell. Rough estimation of computational resources provided in Table 1 shows that it would require more than 70 million core hours of calculations on a typical modern CPU to cover this subset of materials. Given medium-scale HPC computing resources available, we were able to calculate materials with up to 120 of atoms in the unit cell, which have led to 6461 database entries at the time of writing the paper. DFT calculations for the materials with larger unit cells and other carbon-based structures from the rest of the journals are in progress.

**Table 1:** Summary of the input crystallographic data and rough estimation of the computational resources required to complete DFT calculations for the 37,941 organic compounds from four experimental organic chemistry journals contained within the COD database. Core hours (c×h) are estimated based on the actual computational time of self-consistency calculations followed by density of states and band structure calculations on a single-core Intel Xeon 2.2 GHz assuming $O(N_{\text{atoms}}^{2}\log N_{\text{atoms}})$ complexity of the DFT algorithm.

Class	*N*_atoms_	*N* mat. (%)	c×h per mat. mean	c×h total
Small	≤120	6,363 (16.8%)	95	610K
Medium	121–250	16,052 (42.3%)	430	7,000K
Large	251–500	11,598 (30.6%)	1,735	20,000K
Extra large	≥501	3,928 (10.3%)	11,070	43,000K
**Total**		**37,941**		**70,610K**

### Electronic structure calculations

CIF files from the COD database were transformed into input files for the Vienna Ab initio Simulation Package (VASP) [8, 27, 47] by applying the Pymatgen package [41]. For the DFT-based calculations, the projector augmented wave method [48–51] was applied as implemented in VASP and Quantum ESPRESSO [4]. The exchange-correlation functional was approximated by the generalized gradient approximation (GGA) according to Perdew, Burke and Ernzerhof [52]. Within VASP, the precision flag was set to “Normal” and therefore the energy cut-off is given by the maximum of the specified maxima within the POTCAR files. For example for carbon, this value is given by 400 eV. To properly describe the influence of transition metal elements, the calculations were performed spin-polarized. The provided structural information were kept and no further relaxation was considered. For the integration in $\vec{k}$-space, a 6 × 6 × 6 Γ-centered Monkhorst-Pack grid [53] was chosen for the self-consistent cycle. The $\vec{k}$-path for the band structure calculations was automatically generated by the Pymatgen package.

### Database implementation and version control system

The OMDB database is implemented as an open-access database available online at http://omdb.diracmaterials.org. Both the website’s back and front ends were implemented using the PHP language. Additionally, for the front end, the JavaScript language was used. The core of the OMDB is a MySQL database, where all information about materials from the CIF files (e.g. chemical formula, crystal lattice parameters and symmetry group) as well as the output of the DFT calculations (electronic band structures and density of states) are stored. Extended outputs of DFT calculations (e.g., charge distribution, magnetization, orbital projected density of states) for each material are stored in the server’s file system. Every material has a unique OMDB identifier assigned. Furthermore, the COD identifier is kept (if available) to maintain consistency with the COD database.

We use Git [54] to keep track of the development of the database as it represents a widely used version control software. Hence, it is possible to recover a complete history of all modifications of any database entry. Related changes history for each entry is shown on the material’s information page.

## Results

### Database user interface

The user interface and functionality of the OMDB website have been developed in the style of the functionality of the COD database. It allows users to browse through all database entries or particular previously data-mined groups of materials, for example metals or semiconductors. The website also provides a basic search mechanism, where the user can specify full or partial chemical formula, chemical name or symmetry group of interest to retrieve a list of relevant materials. The OMDB also provides a more advanced electronic band structure search, which is described in the following subsection.

**Fig 3:**
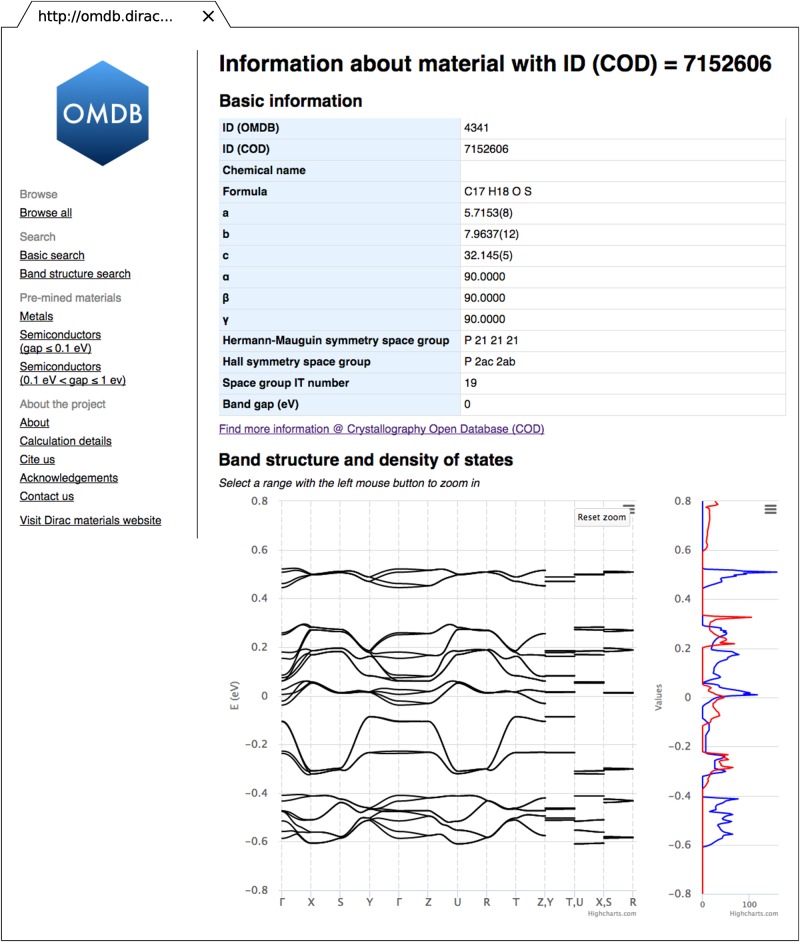
Web interface of the OMDB database. A web page with basic crystallographic information about a material, interactive band structure and density of states plots.

The information page for a selected material (Fig 3) shows basic information about its crystal structure followed by the link to the COD entry if available. It also provides interactive electronic band structure and density of states plots implemented by using the Highcharts JavaScript library [55]. It allows users to zoom in to a specific energy or $\vec{k}$-path range. Furthermore, it is possible to download the plots in one of the popular graphic file formats (PNG, JPEG, PDF or SVG).

### Electronic band structure search

In addition to the basic material retrieval system, the OMDB provides an interface for an advanced band structure search, which can be divided into the two following categories:

1. *“Hard” criteria search*. The database users can provide a rigorous definition of the particular properties the band structure needs to satisfy, for example, presence or absence of a spectral gap of a particular size in a specified energy range.
2. *“Soft” criteria search*. The database users can search for a graphical pattern by making use of a similarity measure, for example, root mean square error (RMSE) or more advanced probability measures [56, 57]. For example, a pattern can specify two crossing straight lines for the search of Dirac materials [58] like graphene or two touching parabolic bands for the search of other semi-metals.

The crucial difference between these two retrieval techniques is that the former completely discards search results which does not satisfy specified search criteria while the latter can only range materials according to some similarity measure, i.e., a single real number. In the latter case, discarding of search results can be based on an essentially subjective threshold value of the similarity measure.

**Fig 4:**
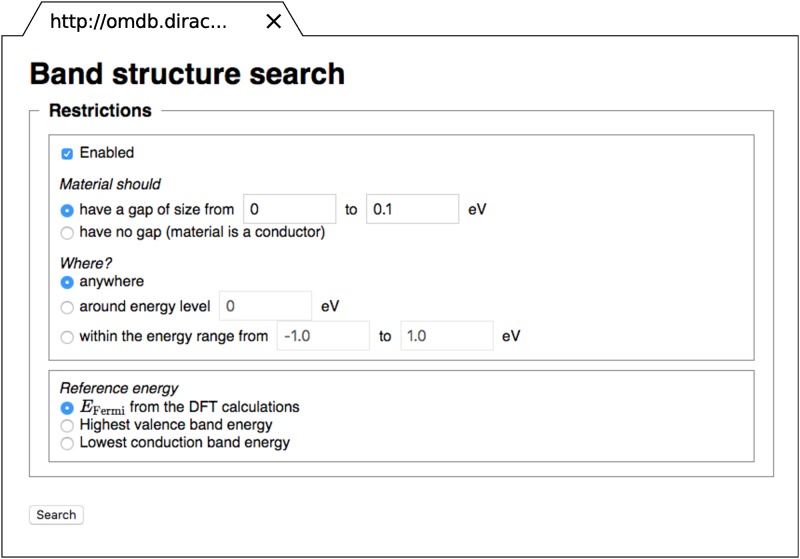
Web interface of the advanced electronic band structure search. Users can search for gap presence/absence of particular size in the energy range.

While the “soft” search technique is only implemented within the offline database version at the moment, the “hard” one is fully functional with acceptable for online usage search execution time. Currently, it provides search possibility for gap presence/absence of particular size in the energy range specified by the user (Fig 4). The other possibilities for this type of search, for instance, the number of electronic bands crossing a particular energy level (which might be important for the discovery of new superconductors), number of electrons or magnetization in the particular energy range, and number of states at the Fermi level, will be implemented in the nearest future. The database users are always encouraged to suggest new search functionality missing within the present version of the database.

## Discussion

**Fig 5:**
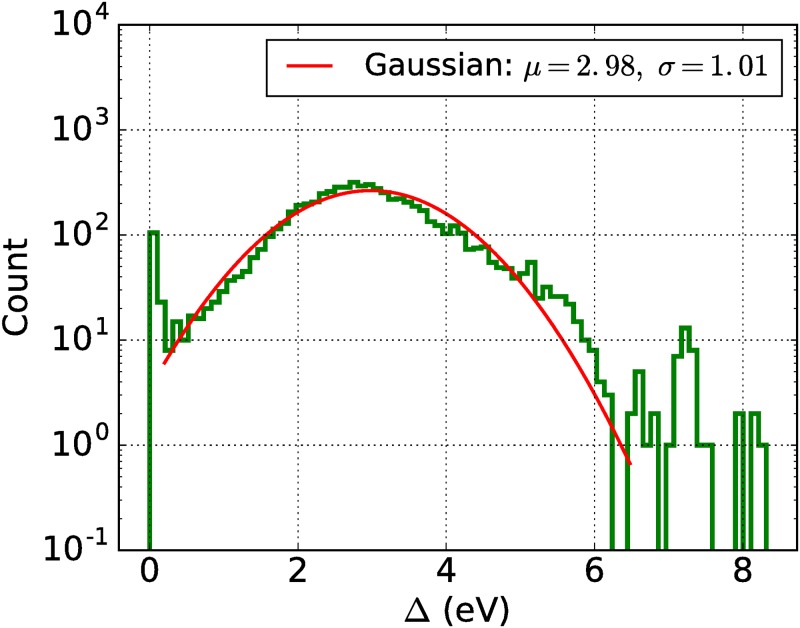
Histogram of the band gap Δ (without distinction between direct and indirect gaps) for 6461 organic materials within the OMDB database. Red solid line denotes Gaussian fit with a mean value *μ* of 2.98 eV and a standard deviation *σ* of 1.01 eV.

As an application of the search tools developed for band structure data mining, we searched for all materials with either zero or small band gap Δ ≤ 1 eV around the Fermi energy. No distinction between direct and indirect band gaps has been made, i.e., Δ was defined as a distance between the minimum energy of the lowest conduction band and the maximum energy of the highest valence band independently of the momentum vector $\vec{k}$. Such materials, metals and semiconductors respectively, are of high practical interest for the organic electronics industry. However, these properties are rarely observed in organic crystals, which are mostly wide-gap insulators [59]. It can also be verified from the histogram of the band gaps of all materials within the OMDB depicted in Fig 5. Remarkably, its bulk shape is close to the (truncated) Gaussian distribution with a mean value of 2.98 eV and a standard deviation of 1.01 eV. Nevertheless, there are a few outliers with a band gap close to zero. In total, by using the implemented OMDB band structure search, 93 suspect materials to be organic metals (Δ = 0 eV), 11 narrow band gap semiconductors (0 < Δ ≤ 0.1 eV) and 151 semiconductors (0.1 < Δ ≤ 1 eV) were identified. The semiconductors as well as metals are tabulated on the OMDB website.

Modern DFT approaches usually fail in accurate band gap estimations as long as no explicit correlation corrections are applied [60]. As mentioned in Materials and Methods, the GGA approximation of the exchange-correlation functional is used, which is known to systematically underestimate bans gaps by about 30–100% [61–63] (see also related discussion on the Materials Project website [64]). Going beyond GGA to improve the accuracy of the DFT band gaps [61, 65–67] and adding experimental data when available is one of the future directions planned for the OMDB. So far, a warning concerning the accuracy of GGA band gaps is shown together with the electronic structures on the website. Nevertheless, the GGA band gap errors can be regarded as statistically systematic in some sense. Particularly, the large number of calculated materials opens up the possibility for a general discussion of trends and features within the electronic structures. It is important to stress that the main goal of the presented database (and most of the other databases containing output from high-throughput DFT calculations) is to provide users with general guidance in the search space.

The application of pattern search algorithms will be available soon within the online version of the website. So far, the offline version has been successfully applied for the search of 3D organic Dirac-point [68] and Dirac-line [69] materials together with an investigation of their topological protection properties for particular crystal symmetry groups.

## Conclusions

We presented the new electronic structure database on organometallics and pure organic materials. The Organic Materials Database (OMDB) currently contains 6461 entries and is accessible via a web-interface at http://omdb.diracmaterials.org. At the current stage, the OMDB database builds the connection between already available structural information, taken from the Crystallography Open Database (COD), with the *ab initio* electronic structure calculations based on the density functional theory (DFT). The implemented structure of the database also allows for an extension beyond the materials contained in the COD database. The presented analysis for the 37,941 materials described in four experimental organic chemistry journals have shown that the number of atoms in their unit cells follows log-normal distribution with the median value of 222 atoms. This relatively large number represents a challenge for high-throughput DFT calculations for organic crystals as the algorithm scales polynomially with the number of atoms. We have roughly estimated that more than 70 millions of core hours of calculations on a typical modern CPU are required to cover this relatively small subset of organic materials. Given medium-scale HPC computational resources, we were able to calculate materials with up to 120 of atoms in the unit cell so far. We plan to extend our calculations to the crystal structures with larger unit cells and materials from other chemical journals in the nearest future.

Although the performed DFT calculations are not fine-tuned to each separate material, the large amount of provided Kohn-Sham band structures and densities of states allows for a general discussion of trends and features within the electronic structures. The core feature of the OMDB is to provide advanced tools aimed for efficient data mining studies of materials with specified electronic target properties. As an example, we discussed the distribution of the band gaps for the calculated materials. Surprisingly, it shows a simple (truncated) Gaussian shape with a mean value of 2.98 eV and a standard deviation of 1.01 eV. Hence, identifying organic metals or semiconductiors is a non-trivial task. The probability of randomly finding a metal using high-throughput DFT calculations is given by 1.4% and of finding a semiconductor with a gap less than 1 eV is less than 2.5%. Although DFT band gaps are usually underestimated, the presented procedure helps to shrink the search space and provide guidance for further theoretical and experimental work. In exchange with the research community, we actively plan to extend the existing OMDB search tools to include broader options related to properties of electronic band structures and density of states.
